# Engineering Secure Self-Adaptive Systems with Bayesian Games

**DOI:** 10.1007/978-3-030-71500-7_7

**Published:** 2021-02-24

**Authors:** Nianyu Li, Mingyue Zhang, Eunsuk Kang, David Garlan

**Affiliations:** 8 Universidad Autónoma de Madrid, Madrid, Spain; 9 University of Twente, Enschede, The Netherlands; 10 Peking University, Beijing, China; 11 Peking University, Beijing, China; 12 Carnegie Mellon University, Pittsburgh, USA; 13 Carnegie Mellon University, Pittsburgh, USA

## Abstract

Security attacks present unique challenges to self-adaptive system design due to the adversarial nature of the environment. Game theory approaches have been explored in security to model malicious behaviors and design reliable defense for the system in a mathematically grounded manner. However, modeling the system as a single player, as done in prior works, is insufficient for the system under partial compromise and for the design of fine-grained defensive strategies where the rest of the system with autonomy can cooperate to mitigate the impact of attacks. To deal with such issues, we propose a new self-adaptive framework incorporating Bayesian game theory and model the defender (i.e., the system) at the granularity of *components*. Under security attacks, the architecture model of the system is translated into a *Bayesian multi-player game*, where each component is explicitly modeled as an independent player while security attacks are encoded as variant types for the components. The optimal defensive strategy for the system is dynamically computed by solving the pure equilibrium (i.e., adaptation response) to achieve the best possible system utility, improving the resiliency of the system against security attacks. We illustrate our approach using an example involving load balancing and a case study on inter-domain routing.

### 1 Introduction

A self-adaptive system is designed to be capable of modifying its structure and behavior at run time in response to changes in its environment and the system itself (e.g., variability in system performance, deployment cost, internal faults, and system availability) [9,12]. One of the major challenges in self-adaptive systems is managing *uncertainty*; i.e., the system should be capable of making appropriate planning decisions despite limited observations about its environment. Achieving *security* in presence of uncertainty is particularly challenging due to the adversarial nature of the environment [17,13]: (1) to avoid detection, a typical attacker may attempt to remain hidden while carrying out its actions, and so accurately estimating its objectives and capabilities can be difficult, and (2) the attacker actively attempts to cause as much harm as possible to the system, and so a typical "average case" analysis may not be appropriate for making optimal defensive decisions [28].

E. Guerra and M. Stoelinga (Eds.): FASE 2021, LNCS 12649, pp. 130–151, 2021.
https://doi.org/10.1007/978-3-030-71500-7_7

Various game-theoretic approaches have been explored in the security community for modeling interactions between the system and attackers as a *game* between a group of *players* (i.e., system and multiple attackers, each as one player) and computing optimal strategies (i.e., Nash Equilibrium) for the system to minimize the impact of possible attacks and improve its resiliency against them [40,15,19,28]. These methods can be used to (1) model adversarial behaviors by malicious attackers [19], and (2) design reliable defense for the system by using underlying incentive mechanisms to balance perceived risks in a mathematically grounded manner [15]. In particular, a type of game-theoretic method called *Bayesian games* [25] is designed to explicitly encode and reason about uncertainty in the information that players have (e.g., partial knowledge about each other's actions and objectives).

Prior works in security that leverage game theory [40,15,19,28] have treated the system as an independent player (i.e., defender) in the game. However, such a monolithic approach that involves abstracting the entire system as a single player might be insufficient for capturing certain practical scenarios, where only one part of the system is compromised while the remaining system components may co-operate each other to mitigate the impact of an on-going attack.

In this paper, we argue that compared to a coarse one-player abstraction of a system, modeling the defender under security attacks at the granularity of *components* is more expressive, in that it allows the design of fine-grained defensive strategies for the system under partial compromise. In particular, we advocate a security modeling approach where an attack is modeled as the anomalous behavior of a system component that deviates from its expected behavior, as an alternative to a conventional approach where attackers themselves are modeled as separate players.

To this end, we propose a novel approach to improving the resiliency of self-adaptive systems against security attacks by leveraging game theory. In particular, we propose a new self-adaptive framework that leverages *multi-players Bayesian games* at the granularity of *components* at the system architecture level. Specifically, in our approach, each major system component is modeled separately as an independent player. Under an attack, one or more components with vulnerabilities might be exploited by an attacker to deliberately perform harmful actions (i.e., turning into a malicious type). Different types of attacks that these components might be subject to are encoded as different *types* of game players, encoding uncertainty in the attack being carried out. The rest of the components are then modeled as forming a coalition to mitigate the impact of the malicious actions by those compromised components.

To perform a security analysis, a model of the system architecture and component attacks are translated into a mathematical Bayesian game structure. Then, the adaptive defensive strategy for the system is dynamically computed by solving a pure equilibrium, to achieve the best possible system utility under all assignments of the components to their possible types (i.e., in the presence of security attacks).

Our main contributions are summarized as follows:

- A self-adaptive framework that incorporates Bayesian game theory to improve the resiliency of the system under potential security attacks;
- An approach to modeling the system under attacks as a multi-player game with potentially uncooperative players at the granularity of components and the use of equilibrium as an optimal adaptation response;
- A demonstration of the applicability of our approach through an example with load-balancing scenarios and a case study involving a network routing application with a proposed dynamic programming algorithm.

## 2 Background

### 2.1 Running Example

As a running example, we adopt Znn.com, a hypothetical news website that has been used as a representative system for the application of self-adaptive systems [10,11]. In a typical workflow, given a request from a client, the web server fetches appropriate content (in form of text) from its back-end database and generates a web page containing a visualization of the text. Furthermore, the system also provides an optional service with multimedia content (e.g., images, videos). This service involves additional computation on the server side, but also brings in more revenue compared to the requests with only text. With $R_M$ and $R_T$ being the revenue, $C_M$ and $C_T$ being the computation of one response to a user request with the media content and with only text content, respectively, we assume that $R_M > R_T > 0$ and $C_M > C_T > 0$.

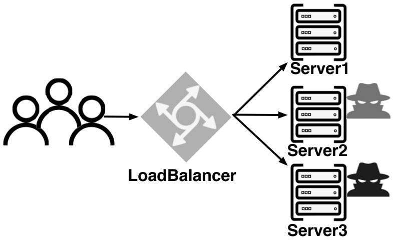

Fig. 1: Running Example.

In order to support multiple servers, a *LoadBalancer* is added to distribute the requests from the users to a pool of servers, as shown in Figure 1. The cost of each server is proportional to its load due to, such as potential high response time since companies such as Amazon, eBay, and Google claim that increased user perceived response time results in revenue loss [33]. To be more specific, the cost per server is denoted by $(S_i - T)^2/K$ where $S_i$ is the current occupied load for server $i$, depending on the request serving mode (i.e., $S_i = D_iC_T$ in text only while $S_i = D_iC_M$ in multi-media mode where $D_i$ is the number of requests distributed to server $i$); $T$ is the threshold beyond which the response time would be affected; $K$ is a constant used to adjust the cost ratio.

The goal of the self-adaptive system is to maximize the difference between revenue and cost.

$$U = R_M x_M + R_T x_T - \sum_{i=1}^{3} (S_i \leq T \ ? \ 0 \ : \ (S_i - T)^2/K) \tag{1}$$

where $x_M$ and $x_T$ are the numbers of responses with media and text content, respectively; the penalty is the sum of the cost for all three servers.

Suppose that some of the servers are vulnerable to various attacks such as password guessing, SQL injection, command injection, etc [1]. The information collected from the web server, however, cannot fully demonstrate its compromise due to, e.g., the deficiencies of scanning tools, but with uncertainty. As shown in the Figure, *Server2* could be potentially attacked with a 20% probability while *Server3* is with a higher probability of 50%. These two servers, if compromised in reality, might perform harmful actions controlled by the attackers to achieve their objectives, rendering the loss of system reward. Here we assume the malicious strategies of simply discarding all the distributed user requests. The reward of attacks is denoted by the system loss, i.e., subtracting the maximum reward the system could achieve from the reward under attacks, leading to a zero-sum game.

#### 2.2 Bayesian Game Theory

*Game theory* is the application of mathematical analysis of individual and cooperative behaviors between players that follow a certain strategy to satisfy their self-interests [21,38]. A *Bayesian* game is a type of game in which players have incomplete information about the other players [25]. For example, a player may not know the exact type (e.g., malicious or good) associated with a unique payoff function of the other players, but instead, have beliefs about these types. These beliefs are represented by a probability distribution over the possible types. More formally, Bayesian games or *incomplete information games* are defined as follows:

**Definition 1.** *A Bayesian game is a tuple* $BG = \langle P, A, \Theta, U, \rho \rangle$

- *A set of* $n$ *players* $P$*;*
- *A set of (joint) actions* $A = A_1 \times ... \times A_n$*, where* $A_i$ *denotes a finite set of actions available to player* $P_i$*;*
- *A set of types for each player* $i : \theta_i \in \Theta_i$*;*
- *A payoff function for each player* $i : u_i(a_1, ..., a_n; \theta_1, ..., \theta_n)$*, determined by the types of all players and actions they choose;*
- *A (joint) probability distribution* $\rho(\theta_1, ..., \theta_n)$ *over types.*

Importantly, throughout the Bayesian games, we assume that the assignment of types to players is private information, while the priori type probability distribution, the action spaces and the payoff functions are assumed to be common knowledge. A player's strategy can be pure (i.e., take a deterministic action) or mixed (i.e., randomly choose an action according to some probability distribution). A strategy for player $i$ is $s_i : \Theta_i \times A_i \to [0, 1]$, and $\forall \theta \in \Theta_i, \sum_{a \in A_i} s_i(a|\theta) = 1$. The strategy is pure if it satisfies that $\forall \theta \in \Theta_i, \exists a \in A_i, s_i(a|\theta) = 1$, also denoted as $s_i : \Theta_i \to A_i$.

**Definition 2.** *(Bayesian Nash Equilibrium Strategy) Given a joint strategy for all players* $\vec{s}^* = [s_1^*, ..., s_n^*]$*,* $\vec{s}^*$ *is the Bayesian Nash equilibrium strategy if for any player* $i$*, it satisfies that:*

$$s_i^* = \arg \max_{s_i \in S(\theta_i)} \sum_{\vec{\theta}_{-i}} \rho(\vec{\theta}_{-i}|\theta_i) \mathbb{E}_{\vec{a}_{-i} \sim \vec{s}^*_{-i}, a_i \sim s_i} [u_i(a_i, \vec{a}_{-i}; \theta_i, \vec{\theta}_{-i})]$$

*where* $\vec{a}_{-i} = [a_1, ..., a_{i-1}, a_{i+1}, ..., a_n]$*,* $\vec{\theta}_{-i} = [\theta_1, ..., \theta_{i-1}, \theta_{i+1}, ..., \theta_n]$*,* $\vec{s}^*_{-i} = [s_1^*, ...s_{i-1}^*, s_{i+1}^*, ..., s_n^*]$*,* $S(\theta_i)$ *is the set of all possible strategies for agent* $i$ *under*

*$\theta_i$, and $\rho(\vec{\theta}_{-i}|\theta_i)$ is the conditional probability representing the player i's belief about other players' types under type $\theta_i$.*
Bayesian Nash equilibrium is a set of strategies, one for each type of player. It is the best strategy that maximizes his or her payoff to other players' equilibrium strategies. In a Nash equilibrium, there is no player who can improve his profit by unilaterally modifying his strategy if the actions of the rest are fixed [25,21].

### 3 Self-Adaptive Framework Incorporating Bayesian Game Theory

Security attacks are usually associated with a high degree of uncertainty where the defender may know little about the identity of the attackers nor fully understand their technical effect on the system. A Bayesian game is a game in which players have incomplete information about the other players, appropriate for modeling and dealing with the attacks with uncertainty. In this section, we propose a new type of self-adaptive framework incorporating Bayesian Game. Adaptation behaviors build on the Nash equilibrium from unexpected attacks and are achieved by elaborating the widely adopted mechanism of the MAPE-K (Monitoring, Analysis, Planning, Execution, Knowledge) loop [27,43], shown in Figure 2.

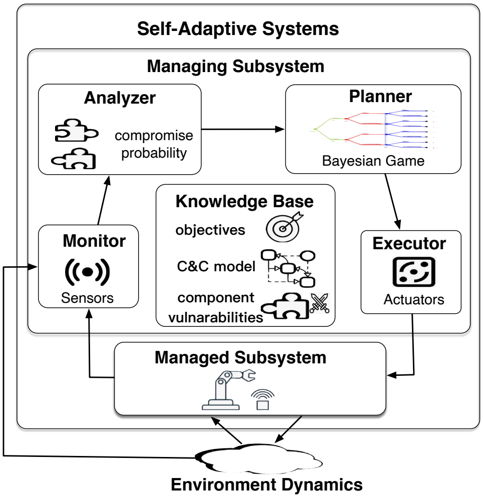

Fig. 2: Self-Adaptive Framework.

**Knowledge.** Knowledge Base requires the system developers or domain experts to specify (1) the component and connector model of the managed subsystem and its action space for each component, (2) system objectives usually defined as the quality attributes quantified by the utility, and (3) component vulnerabilities with potential behavior deviations that can be exploited by the potential attacks. Other necessary information such as the history information of system behaviors and environment information are saved in Knowledge Base and can be updated for the sake of self-adaptation.
**Monitor.** Events generated in the managed subsystem or environment indicating the execution of system actions or natural changes in the environmental factors are received. Monitor gathers and synthesizes the on-going attacks information through sensors and saves information in the Knowledge Base. For our example, events such as plenty of user request loss or command injection can indicate a potential attack on the web server.
**Analyzer.** During speculative analysis, conditions of the environment/managed subsystem representing violations or better satisfaction of goals that can arise based on the input from Monitor are identified. The Analyzer performs analysis and further checks whether certain components are attacked with probabilities; potential deviated malicious actions are identified; the rewards for the attack are estimated, based on the knowledge about component vulnerabilities and system objectives. Such attack probabilities can be analyzed with a statistical combination of all feasible scenarios along with expert judgment [16,24]. A typical example is that both Server2 and Server3 are analyzed to be compromised and discarding user requests with a certain probability, reducing the system utility.

**Planner.** Planner generates a workflow of adaptation actions aiming to counteract violations of system goals or better achieving goals. It consists of one or a set of actions to be enacted by automatically solving the Multi-player Bayesian Game transformed with the input of potential attacks from the Analyzer and architectural model of the managed subsystem along with the system objectives, which is elaborated in Section 4. For each security situation, it generates an equilibrium if one exists as the adaptation to respond to unexpected attacks, or prompts for a change in the design of the system if the violation cannot be handled. Distributing more percentage of a user request to the normal server while decreasing the percentage to those with a high probability of compromise as well as adjusting the fidelity level for servers could be feasible actions for Znn.com Website under security attacks.

**Executor.** During execution, the strategies from the adaptation equilibrium are enacted on the managed subsystem through actuators. Typical examples could be setting the distribution percentage of user percentage in *LoadBalancer* for each server.

In the next part, we focus on planning activity with Bayesian game theory. We assume adequate monitoring in place, sufficient analysis methods on potential attacks with uncertainties based on observation and historical information, as well as an execution environment through which selected adaptation strategies are enacted.

### 4 Bayesian Game Through Model Transformation

In this section, we start by defining the system under attacks and transforming the system architecture and on-going attacks into a component-based multi-player Bayesian game. Solving the game with equilibrium is to find the adaptation strategy. Then, we present the analysis results on our running example.

**Component-based System.** A system component is an independent and replaceable part of a system (e.g., a process, program) that fulfills a clear function in the context of a well-defined architecture. Typical examples are the *LoadBalancer* and servers in Figure 1. Components forming architectural structures affect different quality attributes. For example, quality attributes of user satisfaction (i.e., revenue) and the costs (i.e., penalty) identified in the Znn Website example are influenced by the actions of all four components and characterized as utility functions as shown in Eq.(1) mapping them to utility values.

**Definition 3.** *A system can be formally defined as a tuple* $S = \langle C, A, Q \rangle$.

– *$C$ is a set of components;*
– *$A$ is a set of joint actions $A = A_1 \times ... \times A_n$, where $A_i$ denotes a finite set of actions available to component $i$;*
– *$Q$ is a set of quality attributes a system is interested in; for each $Q_x$, a subset of components $SubC_x \subseteq C$ could contribute to this quality attribute;*

Each component is trying to make the right reaction to maximize the system utility, essentially like a rational player in the game theory. Naturally, a system under normal operation could be viewed as a cooperative game dealing with how coalitions interact. Each component is denoted as an independent player and these interacting components/players form a coalition. For instance, in the running example, the *LoadBalancer* and three servers collaborate to achieve the goals together, i.e., maximizing the system reward with revenue and penalty. Specifically, the *LoadBalancer* should assign more user requests to those servers with low computation usage, like the waiting queue in the bank, while the server should adjust the fidelity level according to its current load. A high load may lead to the text only content to decrease the cost while the server with low usage can provide media content to promote the revenue.

**Modeling Utility as Payoffs.** The payoff among those players is allocated by the utility from quality attributes. It is straightforward for developers to design a system-level payoff function (e.g., the revenue and penalty in Section 2.1). However, due to the different roles of the components and the complex relationship between them, it is complicated and sometimes untraceable to manually design an appropriate component-level payoff function. To solve this problem, we use the *Shapley Value Method*, a solution concept of fairly distributing both gains and costs to several players working in coalition proportional to their marginal contributions [37,36], to automatically decompose the system-level utility into the component-level payoff. *Shapley Value Method* applies primarily in situations when the contributions of each player are unequal, but each player works in cooperation with each other to obtain the payoff. Given the component set $C$, and a system-level utility function $v$, the payoff for a component $i$ is:

$$\phi_i(C, v) = \frac{1}{|C|!} \sum_{C' \subseteq C \setminus \{i\}} |C'|!(|C| - |C'| - 1)![v(C' \cup \{i\}) - v(C')] \quad (2)$$

where $|C|$ is the number of components in the set; $C \setminus \{i\}$ is the set $C$ excluding component $i$; $v(C')$ values the expected system-level utility when the system only consists of the component set $C'$.

The following is a typical example of system utility allocation with the *Shapley Value Method* for the Znn website. To simplify the illustration, we consider the situation where *Server2* and *Server3* are indeed compromised, the *LoadBalancer* chooses the strategy equally distributing user requests to *Server1* and *Server2* (i.e., the requests distributed to *Server1*, *Server2* and *Server3* are 50, 50 and 0 respectively), and *Server1* selects the text only mode. Besides, the total unprocessed requests in the setting are 100, which is assumed to be the full load of a server serving only text, with $R_M = 1.6$, $R_T = 1$,$T = 50$, and $K = 25$ in Eq.(1). The computation capacity of a unit of text and media is 1 and 1.4 (i.e., $C_M$ and $C_T$) respectively. Thus, the system utility in this situation is $U_{system} = 50$ (i.e., $50 \times 1 - (50 \times 1 - 50)^2/25$ with the remaining 50 requests discarded by malicious *Server2*). The *cooperative* player set consisting of *LoadBalancer* and *Server1* share this utility while *Server2* and *Server3* fight on behalf of the attacks' interests, thus not being considered in the coalition neither allocated the payoff from the system utility.

Based on Eq.(2), we need the following two cases of coalitions for Shapley Value calculation: (1) If there is only the *LoadBalancer* without *Server1* in the coalition, the utility of the system $U_{LoadBalancer}$ is 0 due to no requests process from *Server1* neither from malicious *Server2*; (2) If there is only *Server1* without *LoadBalancer* distributing user requests, the requests are randomly passed among three servers, i.e., the requests distributed to *Server1*, *Server2* and *Server3* are 34, 33 and 33 respectively, and the utility of the system for this coalition $U_{server1}$ is 34 (i.e., $34 \times 1 - 0$). This is because malicious *Server2* and *Server3* do not return any feedback. As a result, $\phi_{LoadBalancer}(C, v) = 1/2(U_{system} - U_{server1} + U_{loadbalancer}) = 8$ and $\phi_{Server1}(C, v) = 1/2(U_{system} - U_{LoadBalancer} + U_{server1}) = 42$. Therefore,the payoff to player *LoadBalancer* and *Server1* are 8 and 42 respectively. Meanwhile, attacks' utility, the difference between system utility and the highest utility the system could achieve without attacks (i.e., equally distributing user requests to three servers and each server choosing multi-media mode in this setting with value $160 = 100 \times 1.6 - 0$) is equally divided for two malicious players. In other words, both *Server2* and *Server3* is allocated payoff 55 = (160-50)/2. Following the aforementioned allocation process, each player obtains a unique payoff under different attack situations and strategies from the *Shapley Value Method* based on their roles contributing to marginal system utility.

**Component-based Attacks.** A system under security attacks is also defined as a tuple $SAS = \langle C, A, Q, ATT \rangle$. Instead of modeling an attacker or several attackers with possible complex behaviors over different parts of the system, we model the on-going attacks $ATT$ the system is enduring at the component level since the vulnerabilities of the components as well as their potential behavior deviations are comparatively easy to observe. $ATT$ can be obtained by synthesizing the information from Monitor and Analyzer as described in Section 3.

**Definition 4.** *The security attacks on the system is formally defined as a tuple* $ATT = \langle C_{att}, A_{att}, P_{att}, R_{att} \rangle$.

- $C_{att}$ *is the set of components affected by the attacks;*
- $A_{att} = A_{att1} \times ... \times A_{attm}$ *where* $A_{atti}$ *denotes the set of actions controlled by attacks on compromised component i;*
- $P_{att} = \{p_1, ..., p_m\}$ *is a set of probability where* $p_i$ *is the probability of component i being successfully compromised;*
- $R_{att}$ *is the reward for attacks.*

**Translation into a Bayesian game** With the definition of the system on the component level and the definition of the attacks $ATT$, a system under security attacks is converted into a non-cooperative Bayesian game by the following steps:

1. Each component in the system $c \in C$, such as *LoadBalancer* and three servers in the running example, is separately modeled as an independent player;
2. The components potentially affected by attacks $C_{att} \subseteq C$ is associated with two types (e.g., *Server2* and *Server3* can be *normal* or *malicious* in the simplified Znn website scenario) while the remaining components $C - C_{att}$, i.e., *LoadBalancer* and *Server1*, are deterministic in *normal* type;
3. The probability distribution for a player $i$ over two types is $\rho(p_i, 1 - p_i)$ as defined in $P_{att}$. One typical example for *Server2* is $\rho(0.8, 0.2)$ and for *Server3* $\rho(0.5, 0.5)$;
4. The action space of player $i$ under security attacks is the union of both its normal actions and those malicious actions controlled by attacks (i.e., $A_i \cup A_{atti}$). *Server2* can serve user requests either with text only or multimedia content as a normal player, or maliciously discard them with the intention of attacks;
5. The payoff for players in normal type is allocated with system utility by the *Shapley Value Method*, while components in malicious type performing harmful actions is assigned with utility the on-going attacks obtain by achieving their own goals. This assignment could be simple average distribution or *Shapley Value Method* if the malicious players are treated as another coalition;
6. The game constructed is put into a game solver, to find a Nash equilibrium, which, in essence, is the best reaction for the system to potential attacks.

Note that this definition can be easily extended for the situation where a component is simultaneously compromised by different attackers with multiple types. Besides, the game solver we adopted in this work is *Gambit* [35], a collection of tools for building game models, computing game equilibrium and analyzing game results, to efficiently model the Bayesian game translated by the above steps and automatically figure out the equilibrium strategy as the adaptation response.

#### 4.1 Analysis Results for Znn.com Example

In this subsection, we demonstrate how our approach can produce adaptation decisions under security attacks for Znn website to enhance the system utility. In particular, we exploit the Bayesian game model by following the aforementioned steps and generate the equilibrium. To explore different attack scenarios, we statically analyze a discretized region of the state space, which is projected over two dimensions that vary the malicious probability (i.e., $probability\_S2$ and $probability\_S3$) of *Server2* and *Server3* respectively (with values in the range [0, 1]). Each state of the discrete set requires a solution of the game with the Nash Equilibrium that quantifies the best utility the system could obtain. The experiment takes less than one minute to generate all the results, as shown in Figure 3, and for each state, the solution generation time is negligible. To set up the experiment, we assume there are 100 user requests - the maximum load of a server in text only mode - with $R_M = 1.6$, $R_T = 1$, $x_M = 1.4$, $x_T = 1$, $T = 50$, and $X = 25$ in Eq.( 1). Additionally, we adopt the probabilistic model checking method as the benchmark [11,7,32] and compare our Bayesian Game theory method with it in terms of the system utility.

Figure 3 (a) illustrates the percentage of user requests distributed to *Server1* from the strategy for the *LoadBalancer* in equilibrium. As expected, the percentage of *Server1* increases progressively with the increasing malicious probability of *Server2* and *Server3* as more user requests are supposed to be processed by a server under normal operation. In particular, we observed that the user percentage is around one third when both *Server2* and *Server3* are functioning normally (i.e., both $probability\_S2$ and $probability\_S3$ are 0), with *LoadBalancer* equally delivering the user requests since none of the servers is compromised. Moreover, the percentage for *Server1* reaches around 84% when the other two servers are fully compromised. In this situation, *LoadBalancer* does not deliver all user requests to *Server1*; otherwise *Server1* may be overloaded with the increasing costs due to high response time which in turn outweigh its benefits of request processing.

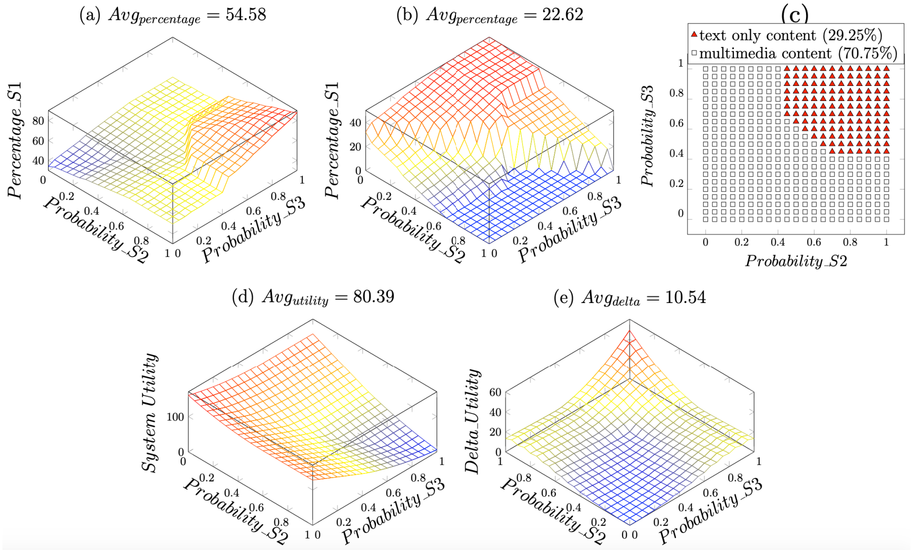

Fig. 3: Results for Znn Website: (a) percentage of user requests to *Server1*; (b) percentage of user requests to *Server2*; (c) strategies for *Server1*; (d) system utility with game theory approach; (e) delta utility between Bayesian game theory approach and probabilistic model checking approach.

Figure 3 (b) describes the percentage of user request that *LoadBalancer* delivers to *Server2* in the equilibrium. We can also observe that user requests to *Server2* are negatively proportional to its malicious probability. Particularly, user requests are 50 when probability $probability\_S2$ is 0 while *Server3* is fully malicious (i.e., $probability\_S3$=1) where *LoadBalancer* should equally distribute the user request to both *Server1* and *Server2*. Figure 3 (c) presents the strategy in equilibrium for *Server1*. The states in which text content is provided are indicated by red triangles, whereas the multimedia strategies for *Server1* are denoted by white rectangles. As we can see, red points are in the upper right corner where malicious probabilities of *Server2* and *Server3* are greater than 50%, which means that they are very likely compromised. Therefore, *LoadBalancer* distributes as many user requests as possible to *Server1*, thus *Server1* choosing to provide text only content in avoid of overloading. Otherwise, *Server1* can provide multimedia content in less load condition to promote user satisfaction with higher revenue.

Figure 3 (d) illustrates the maximum utility the system can achieve under various attack situations. In particular, we observe that the utility reaches around 160 when all three servers are cooperative and is progressively decreased with the increasing malicious probability of *Server2* and *Server3*. This is consistent with the fact that the system utility is deteriorated under security attack. To compare the system utility in game theory with existing methods, we adopt probabilistic model checking [29] as the comparison standard to formally model the running example and synthesize the adaptation strategy maximizing its expectation of the utility by reasoning about reward-based properties [11,7,32]. Figure 3 (e) presents the delta between two approaches (i.e., system utility with game theory approach minus the utility with the probabilistic model checking approach). Without security attacks, the adaptation decision generated by the two approaches achieve the same utility. However, with the increasing malicious probability of *Server2* and *Server3*, game theory approach outperforms, providing the better response to make up for the utility loss due to security attack, and the average delta is 10.54, i.e., 15 percent outperforming with the average utility 80.39 achieved by game theory.

### 5 Evaluation – Routing Games

To evaluate our approach and assess its applicability for validation, we consider a case study on an interdomain routing application. We first define the game (Section 5.1) and propose a dynamic programming algorithm to solve the equilibrium by decomposing the problem into smaller and tractable sub games (Section 5.2). The results are present (Section 5.3) with a sensitivity analysis, illustrating how the system can choose a robust strategy effective for a range of threat landscapes, and a utility analysis by quantifying the defender's utility with Bayesian game compared to a greedy solution within the security context.

A routing system is usually composed of smaller networks called nodes as shown in Figure 4. Since not all nodes are directly connected, packets often have to traverse several nodes and the task of ensuring connectivity between nodes is called interdomain routing [30,31]. Each node could be owned by economic entities (Microsoft, AT&T, etc.) and might be compromised by the attacker at any time. Therefore, it is natural to consider interdomain routing from a game-theoretic point of view. Specifically, game players are source nodes located on a network, aiming to send a package (i.e., starting at $N1$) to a unique destination node (i.e., $N5$). The interaction between players is dynamic and complex – asynchronous, sequential, and based on partial information - and the best strategy for each player as the adaptation response is updated as needed.

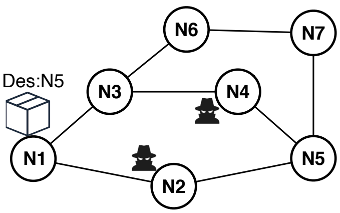

Fig. 4: Routing Scenario.

#### 5.1 Game Definition for Interdomain Routing

The interdomain routing system is described below with the component-based definition.

- The components set for the interdomain routing is $C = \{N1, N2, ..., N7\}$;
- The action space for each node is to deliver the package at hand to its neighboring nodes. Typical example is $A_{N1} = \{toN2,\ toN3\}$;
- The only quality attribute this network needs to be concerned with is the time delivering the package to its destination as we assume there is no case of package loss. Specifically, we consider the delivery time is proportional to the distance denoted by hops between nodes. Its utility function is encoded using a formula that enables the quantification of the utility of a given state and defined as $U_{system} = 10 - \#hops$. Usually, the longer time, the lower utility and the maximum utility system could achieve under normal operations for this network is 8 with two hops $\langle N1\ N2\ N5 \rangle$;

Currently, $N2$ and $N4$ are analyzed to be potentially attacked based on the historical package delivery record, deliberately sending the package in the opposite direction, extending the delivery time. The game definition with the security attacks is summarized below.

- The player set for the game is $C = \{N1, N2, ..., N7\}$. The set of affected components by the attack includes $N2$ and $N4$, i.e., $C_{att} = \{N2,\ N4\}$;
- The action set for all players, including malicious ones controlled by attacks, is delivering the package to its neighboring nodes.
- The set of types for potential attacked component node includes "normal" and "malicious" (i.e., $\theta_{N2} \in \{normal,\ malicious\}$, $\theta_{N4} \in \{normal,\ malicious\}$).
- The payoff for all the normal players is allocated by the system utility with the *Shapley Value Method* (i.e., $U_{system} \div |normal\ players|$, equally allocated in this case since all of the nodes in this network is not cut vertex with the same importance). For example. each node is awarded 8/7 if none of them is attacked. The utility for the ongoing attacks on two components is the utility loss from the system's best response without attack, rendering a case of zero-sum game.
- The probability distribution for both component $N2$ and $N4$ could be, e.g., 50%/50% split (i.e., $\rho_{N2,N4}(normal,\ malicious) = (0.5, 0.5)$.

#### 5.2 Dynamic Programming Algorithm

In practice, a network might be complex and each node could have hundreds of neighboring nodes. It is impractical to directly build a game tree, in the component level with a large number of players (each with a massive action set), and solve such a network in a reasonable time. To deal with the complexity of network nature, we propose an algorithm inspired by dynamic programming to effectively solve the generated Bayesian game for this class of routing problems.

The algorithm 1 for routing game has as input a routing network $N$ – consisting of a starting point $s$ of package delivery and a destination point $d$. To carry out dynamic programming, the algorithm uses a set $subG$ to store the set of nodes which have been processed with their best reactive strategy. $subG$ is initialized as an empty set (line 1) and added with node $d$ (line 2) since $d$ does not need the strategy to transmit the package. The algorithm starts by iterating all the nodes in the distance $disValue$ (line 5), initialized by 1 (line 3). For example, $N2$, $N4$ and $N7$ are qualified in the first iteration. Each node is checked whether it is potentially attacked (i.e., $uncertain(n)$ in line 6). For those uncertain nodes (e.g., $N2$ and $N4$), they might affect the strategy of their prior nodes (line 7) (e.g., $N1$ and $N3$), which shall be added to $todoS$ (line 8), to be processed to update their strategy due to its neighboring uncertainty. A typical example is that node $N3$ might trade off the delivery between $N4$ and $N6$ even though $N4$ is in the shortest path from $N3$ to $N5$, however, could deliberately send the package back controlled by the attack. If the node is not in $todoS$ to be updated (line 11), it is directly added to the $setG$ (line 12) as the best strategy for such benign node is passing the package down to its adjacent node along the shortest path. In this routing scenario, $N2$, $N4$ and $N7$ is added to $subG$ as their strategies in equilibrium with normal type is easily determined.

After iterating all the nodes in $disValue$ 1, each node in $todoS$ (line 15) is checked whether it satisfies the condition (line 16) where all its neighboring nodes (i.e., $i \in adj(n)$ ) closer to destination (i.e., $dis(i,d) == dis(n) - 1$) have been solved with their best strategies (i.e., in $subG$), to build a sub-game. As shown in the example, though both $N1$ and $N3$ are prior to an uncertain node, their strategy update is postponed as $N6$ is not in $subG$ yet, which affects the sub-game generation for $N3$, in turn delaying the sub-game construction for $N1$.

An exemplified subgame construction (line 17) starting from $N3$ is illustrated in Fig 5 when all conditions are satisfied. The stochastic behavior of those potentially compromised nodes can be modeled by introducing a nature (or chance player), who moves according to the probability distribution (e.g., 50%/50% split), randomly determining whether attacks on $N2$ and $N4$ are successful. Then, $N3$ can choose an action passing to the one from the set of its adjacent nodes, i.e., $N6$ or $N4$. Here, $N3$ is a normal node aware of that the package is transmitted from $N1$ and it is not necessary to consider a rollback to $N1$. The game is ended after $N3$'s action as we can prune the following branches: 1) to $N6$, the remaining route sequence is $N7$ and $N5$ by default as their best strategy have been solved (i.e., $N6$ delivers the package to $N7$, which in turn forwards to $N5$); 2) to $N4$, with $N4$ forwarding to $N5$ if it is normal while backing to $N3$ in malicious type. When the game terminates, each player gets a unique payoff following different branches. As

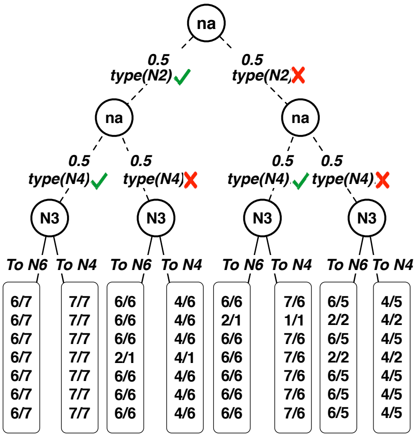

Fig. 5: Sub-Game for N3.

**Algorithm 1** Dynamic Programming Algorithm to Solve Routing Game.

```
1:  setG ⇐ ∅
2:  addNode(d, setG)
3:  disValue ⇐ 1
4:  repeat
5:     for all n ∈ N  and  dis(n, d) == disValue do
6:        if uncertain(n) == true then
7:           for all n_p ∈ adj(n)  and  dis(n_p, d) == disValue + 1 do
8:              addNode(n_p, todoS)
9:           end for
10:       end if
11:       if n ∉ todoS then
12:          addNode(n, setG)
13:       end if
14:    end for
15:    for all n ∈ todoS do
16:       if ∀i ∈ adj(n) and dis(i, d) == dis(n) − 1 and i ∈ sutG then
17:          gambitTree ⇐ buildGame(n, d)
18:          equilibria ← solve(gamebitTree)
19:          removeNode(n, todoS)
20:          addNode(n, setG)
21:       end if
22:    end for
23:    disValue ⇐ disValue + 1
24: until s ∈ subG
```

shown in the left most rectangle all the players (including $N2$ and $N4$ as they are benign collaborating nodes) equally share the system utility value 6 with 3 hops from $N3$ to $N5$ plus the shortest path from $N1$ to $N3$. However, on the rightmost branch, only five players ruling out $N2$ and $N4$ is allocated with the system utility 4. The system utility is resulting from 6 hops if $N3$ decides to deliver the package to $N4$ as the nature problematically chooses the malicious type for $N4$, which sends the package back to $N3$ to maximize the attack's utility. Once $N3$ receives the package from $N4$, it redelivers the package to $N6$ because $N3$ as a good player does not repeatedly send it back. To this end, $N2$ and $N4$ is uniformly allocated the delta (i.e., 4) between the utility system obtained (i.e., 4) and the maximum utility system could obtain (i.e., 8) as the payoff. The payoff of the remaining branches can also be calculated accordingly.

After that, a pure Nash equilibrium is generated by solving this sub-game (line 18) with Gambit software tools [35], and the best strategy for the node is updated according to the equilibrium. By solving the sub-game for $N3$, the strategy for $N3$ in the equilibrium is to deliver the package to $N6$, as the potential detriment on delayed delivery time to $N4$ due to attacks is greater than its comparative advantage of the shortest path. Thus, this node with the solved strategy is removed from *todoS* (line 19) and absorbed in *setG* (line 21). Once all the nodes in the distance of *disValue* from the destination have been iterated and all the nodes in *todoS* satisfying conditions are computed for their best strategy, the algorithm increment the value of *disValue* one unit (line 23) and continue, until the starting point $s$ is in the set *setG* (line 24).

#### 5.3 Experiment Setup & Results

We demonstrate how our Bayesian game approach combined with the proposed dynamic programming algorithm can produce adaptation decisions about how to forward packages for each node in the routing example. Similar to the experiment results found on the Znn website, we statically analyzed a discretized region of the state space which represented different attack scenarios (i.e., malicious probability of $N2$ and $N4$). The entire experiment setup of the network structure is exactly shown in Figure 4. In addition, we also adopted a greedy algorithm for this routing application as the benchmark, and compared the system utility between these two approaches to demonstrate the superiority of game theory under security attacks. The experiment for the whole state space with Bayesian approach takes less than one minute and the solution generation time for each state is negligible.

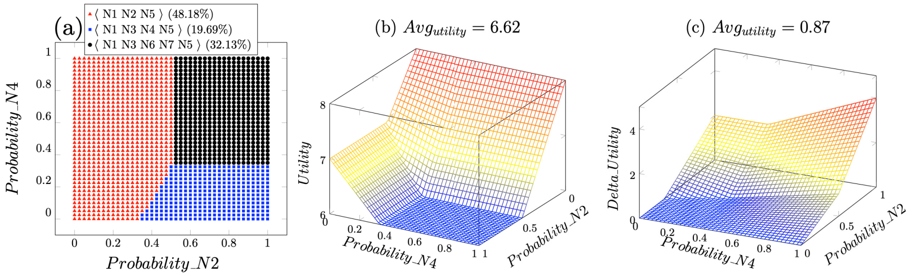

Fig. 6: Results for interdomain route example: (a) Expected route in equilibrium; (b) System utility with game theory approach; (c) Delta between system utility from game theory approach and utility from greedy algorithm.

Figure 6 (a) presents the results of the strategy selection (i.e., expected package sequence) over two dimensions that correspond to the malicious probability of $N2$ and $N4$, respectively. Red triangle points denote that the strategy for $N1$ is $N2$, extending the range of $Probability\_N2$ to around [0, 0.50]. This is because when the chance of $N2$ coming under attack is less than 0.50, $N1$ should pass the package to $N2$, since $N2$ is in the shortest path to the destination; otherwise, $N1$ delivers the package to $N3$. Similarly, when the malicious probability of $N4$ is less than 0.35, the strategy for $N3$ reaching equilibrium is to deliver the package to $N4$ (i.e., blue square points), since the benefits of a short delivery time outweigh the potential detriment. For the remaining situations denoted by the black circle points, $N1$ passes the package to $N3$, which in turn forwards it to $N6$.

Figure 6 (b) describes the utility the system could obtain for the attacked components' equilibrium strategies. As expected, when the $Probability\_N2$ is greater than 50% and $Probability\_N4$ greater than 35% (i.e., black circle points in Figure 6 (a)), the utility system can gain is 6 as there are 4 hops in the expected sequence $\langle N1\ N3\ N6\ N7\ \ N5\rangle$). This plot also shows that the system utility increases progressively with decreasing probability of the compromised $N2$ and $N4$. When the $probability\_N2$ is 0, the expected utility increases to 8 (i.e., two hops in $\langle N1\ N2\ N5\rangle$). Similarly, the utility reaches 7 with $probability\_N4$ 0 (i.e., three hops in $\langle N1\ N3\ N4\ N5\rangle$).

Furthermore, we adopted a baseline that generates strategies for each node in a non-repeating fashion, passing the package to the adjacent node along the shortest path to the destination. The aim of this was to compare the utility between two different approaches dealing with security attacks. For the network as shown in Figure 4, the baseline firstly picks up the shortest path sequence $\langle N1\ N2\ N5\rangle$. If $N2$ is compromised and sends the package back, $N1$ redelivers it to $N3$ instead of $N2$ since the package is received from $N2$. The system utility for the greedy algorithm is the expected value, the weighted average of utility for paths in different attack situations. Figure 6 (c) shows the delta between the utility produced by our game theory method and the utility produced by the baseline. During security attacks, we can see that the utility from the game theory approach is always higher than the greedy approach under security attacks. The delta is much more noticeable, especially in the situations where $N2$ and $N4$ are highly likely to be compromised (i.e., $Probability\_N2$ and $Probability\_N4$ close to 1). This is because game theory approaches can help the defenders to trade off the gains and losses due to perceived risks.

In summary, based on the preliminary results of our experiment, our game theory approach in the component level applies to self-adaptive applications. To adopt our approach, attacks information, such as various types with probabilities as well as its payoff, shall be provided from the *Analyzer*, to construct a Bayesian game based on system architectural structures. The results have also shown that game theory can enhance the performance of the system, especially when a potential attack is more likely to happen. In these situations, game theory approaches could help the defenders balance perceived risks by using underlying incentive mechanisms, and figure out the best response as the adaptation to be executed on the network using proven mathematics. Besides, our proposed dynamic programming algorithm is specific to this kind of application to optimize the game solving. Another potential application is the multi-agent finding (MAPF) problem where a spatial position in a path can be viewed as a node in the network [39,3]. Other optimization techniques might be adopted or customized for different applications with complicated game structures.

### 6 Related Work

Self-adaptive systems under security attacks need to make adaptation decisions as a response to a detected threat or to deviations from security goals and requirements [18]. Lorenzoli et al. [34] proposed a technique that could observe values at relevant program points and identified the execution contexts leading to a software failure so that mechanisms can be enabled for preventing future occurrences of failures of the same type. Bailey et al. [4] generated Role Based Access Control (RBAC) models to provide assurances for adaptations against insider threats. RBAC technique was also applied to cloud computing environment to provide appropriate security services according to the security level and dynamic changes of the common resources [44]. Tsigkanos et al. [41] explored the use of Bigraphical Reactive Systems to perform speculative threat analysis through model checking. Burmester et al. [5] described a threat model to incorporate typical characteristics of systems, such as survivability to abnormal behavior and possibility to recover after critically vulnerable states are reached. Dimkov et al. [14] discussed insider threats that span physical, cyber and social domains and present a framework Portunes integrating all three security domains to describe attacks. Nashif et al. [2] presented a multi-level intrusion detection system to detect network attacks within three levels of granularities and proactively protected against them by employing a fusion decision algorithm. Although, there are many different ways of dealing with security attacks in self-adaptive systems, it is notable that the application of game theory, with the characteristic of modeling the adversarial nature of security attacks and designing reliable defense with proven mathematics, has not gained the deserved attention.

Different sorts of games have been employed to study the actions of the defender and attacker. Dijk et al. [42] presented a two-player game that reasons about security scenarios where an attacker with uncertainty about its actions may periodically gain full control of an asset, with each side trying to maintain control as much as possible. An extension work by Farhang et al. [19] explicitly modeled the information gains for the attackers as they control assets, improving attacker's capability. Based on these work, Kinneer et al. [28] additionally considered multiple attacker types with different goals and capabilities by Bayesian Game. Instead of modeling the attackers as independent players, our work models the attacks on the component level, focusing on the defender modeling at the architecture level and possible deviations of component behaviors. Cámara et al. [6,8] adopted a game-theoretic perspective and model the system as turn-based stochastic multi-player games between different players where players can either cooperate to achieve the same goal or compete to achieve their own goals. In addition, Glazier et al. [23] used game-based approach to automatically reason and synthesize strategies for meta-manager by explicitly considering alternate potential future state, thus improving the performance of a collection of autonomic systems against a defined quality objective. Though, some of these existing works concern about competitive behaviors in a system when some components cannot be controlled and even behave according to conflicting goals with respect to other components in the system. None of them, to the best of our knowledge, proposed to model the Bayesian game in an architecture/component level and captured multiple attacks as component's variant types as well as the uncertainty due to unsuccessful compromise.

Game theory is also increasingly applied to network security. Frigault et al. [20] measured the network security in a dynamic environment with dynamic Bayesian networks-based model to incorporate temporal factors. Charles et al. [26] developed a packet forwarding game model under imperfect private monitoring. Their equilibria rely on the probability of cooperation after observing a defection, similar to our routing games in the evaluation. However, they looked at this problem from the perspective of network nodes, without considering the situation of being attacked and how to allocate rewards from the system utility for multiple components from the architecture perspective as illustrated in this work.

### 7 Conclusion and Future Work

In this paper, we have proposed a new framework for self-adaptive systems by adopting Bayesian game theory and modeled the system under security attacks as a multi-player game. An optimal adaptation strategy for responding to attacks is generated by computing the equilibrium to the game. One limitation is that we validate our approach on a simulated rather than an actual system, and we plan to further evaluate the applicability and scalability of the approach using case studies involving real systems. A second limitation is the simplification of the amount of uncertainty, such as restricting the number of component types under attacks and assuming the payoffs with zero-sum game, which might be more complex in the real world security landscape. Rather, we attempted to convey the idea of transforming the system architecture consisting of multiple components under attacks into a Bayesian game. While the equilibrium is sensitive to the probability distribution over types (i.e., malicious probability), sensitivity analysis are useful when the probability cannot be determined by the analysis with precision but lies within a known range. In addition, modeling attacks on component level, though more monitorable and easy to handle, cannot depict those attacks with highly motivated and capable adversaries willing to devote significant time and continuous attack to facilitate their malicious goals, known as advanced persistent threats (APTs) [28].

Moreover, we adopt pure equilibrium as the adaptation response. However, in practice, there will likely be multiple equilibria and no guarantee of uniqueness. While this is an area for future work, one possible way to overcome this is to choose the equilibrium with highest utility for the system. Another limitation, and a topic for future work, is that mixed equilibrium is another common solution for game theory. Its interpretation on system behaviors could be various and allows generation of different types of defense strategies for the system, which can be explored for different applications. For example, if the mixed strategy for N1 in routing game is choosing N2 and N3 in 50%/50% split as shown in Figure 4, we can consider that N1 may equally distribute its packages to N2 and N3 if multiple packages exist, or deliver its packages to N3 for the current time and to N2 next time. Also, the Bayesian games for these two examples were manually created by following the framework into the input language of the Gambit tool, to solve the equilibrium. In future, we are planning to construct the game in an automated way by supporting an architecture description interchange language, such as Acme [22].

### Acknowledgements

The research is partially supported by the National Natural Science Foundation of China under Grant Nos. 61620106007 and 61751210, award N00014172899 from the Office of Naval Research and the NSA under Award No. H9823018D0008.
